# Control of prostate cancer associated with withdrawal of a supplement containing folic acid, L-methyltetrahydrofolate and vitamin B_12_: a case report

**DOI:** 10.1186/1752-1947-5-413

**Published:** 2011-08-25

**Authors:** Glenn Tisman, April Garcia

**Affiliations:** 1 Whittier Cancer Research Building, 13025 Bailey Street, Whittier, CA 90601, USA

## Abstract

**Introduction:**

This is the first report of possible direct stimulation of hormone-resistant prostate cancer or interference of docetaxel cytotoxicity of prostate cancer in a patient with biochemical relapse of prostatic-specific antigen. This observation is of clinical and metabolic importance, especially at a time when more than 80 countries have fortified food supplies with folic acid and some contemplate further fortification with vitamin B_12_.

**Case presentation:**

Our patient is a 71-year-old Caucasian man who had been diagnosed in 1997 with prostate cancer, stage T1c, and Gleason score 3+4 = 7. His primary treatment included intermittent androgen deprivation therapy including leuprolide + bicalutamide + deutasteride, ketoconazole + hydrocortisone, nilandrone and flutamide to resistance defined as biochemical relapse of PSA. While undergoing docetaxel therapy to treat a continually increasing prostate-specific antigen level, withdrawal of 10 daily doses of a supplement containing 500 μg of vitamin B_12 _as cyanocobalamin, as well as 400 μg of folic acid as pteroylglutamic acid and 400 μg of L-5-methyltetrahydrofolate for a combined total of 800 μg of mixed folates, was associated with a return to a normal serum prostatic-specific antigen level.

**Conclusion:**

This case report illustrates the importance of the effects of supplements containing large amounts of folic acid, L-5-methyltetrahydrofolate, and cyanocobalamin on the metabolism of prostate cancer cells directly and/or B vitamin interference with docetaxel efficacy. Physicians caring for patients with prostate cancer undergoing watchful waiting, hormone therapy, and/or chemotherapy should consider the possible acceleration of tumor growth and/or metastasis and the development of drug resistance associated with supplement ingestion. We describe several pathways of metabolic and epigenetic interactions that could affect the observed changes in serum levels of prostate-specific antigen.

## Introduction

The clinical course of our patient with hormone-refractory or hormone-resistant prostate cancer appears to have been affected by ingestion followed by withdrawal of a vitamin supplement containing a mixture of large amounts of folic acid (FA), L-methyltetrahydrofolate (L-methyl-THF, or folate) and cyanocobalamin (vitamin B_12_). Prior to supplement withdrawal, the patient had been treated with docetaxel for 18 weeks but had a continuous rise in serum prostatic-specific antigen (PSA) levels. Only after withdrawal of the supplement did the patient's elevated serum PSA level return to normal (from 22 ng/mL to 2.08 ng/mL).

### Biological and clinical background

In 1946, Lewisohn *et al*. [1] reported the effects of pteroylglutamic acid (teropterin) and FA (defined as liver *Lactobacillus casei *factor) on mice with spontaneous breast cancer. Careful examination of their results revealed that the newly discovered FA stimulated, in a dose-dependent fashion, the growth and metastasis of spontaneous murine breast tumors and shortened overall survival. Two years later Heinle and Welch [2] reported FA stimulation of chronic myelogenous leukemia (CML) in three patients so inflicted. In 1948, Farber [3] referred to an "acceleration phenomenon" observed while treating 10 children with leukemia with pteroylglutamic acid (diopterin) and teropterin. In 1950, Skipper *et al*. [4] reported that large doses of FA alone and in combination with aminopterin modulated the survival of mice with the transplanted acute Ak4 strain of leukemia. He surmised that FA is a rate-controlling factor in Ak4 leukemia. An excess of FA clearly accelerated the leukemic process, causing the animals to die before untreated controls. Acceleration of CML by vitamin B_12 _in patients with pernicious anemia was reported by Corcino *et al*. in 1971 [5] and Green in 1994 [6]. In 2009, Tisman *et al*. [7] presented evidence for the acceleration of prostate cancer dedifferentiation during vitamin B_12 _depletion and prostate cancer acceleration in response to vitamin B_12 _administration in a patient with localized prostate cancer and pernicious anemia. In 2009, Figueiredo *et al*. [8] reported the results of a large, randomized, controlled clinical trial carried out over 10 years in which a group of men received 1000 μg of oral FA daily. They observed a near tripling of the incidence of prostate cancer compared to controls. Finally, a report by Lawson *et al*. [9] published in 2007 described a direct relationship between prostate cancer stage and multivitamin use. During that period, most multivitamins contained an additional 400 μg of FA.

### Case presentation

Our patient is a 71-year-old Caucasian man who had been diagnosed in 1997 with prostate cancer. His baseline PSA level was 8 ng/mL. All six biopsy cores contained 90% Gleason scores of 3+4 = 7 adenocarcinoma, and peri-neural invasion was observed. The patient's clinical stage was T1c. He elected therapy with intermittent androgen deprivation (IAD) with flutamide, leuprolide, and finasteride. In 2007, after the third cycle of IAD, his PSA level slowly increased into the 3 ng/mL range and his serum testosterone remained < 20 ng/dl. Sequential anti-androgen withdrawal, ketoconazole, diethylstilbestrol, estramustine, and transdermal β-estradiol, along with a trial of low-dose oral cyclophosphamide and capecitabine, all while he was being treated with leuprolide maintenance therapy, were either transiently effective or unsuccessful.

The patient was restaged, with a bone scan and computed tomography yielding only evidence of biochemical PSA relapse. He then received docetaxel 30 mg/m^2 ^for three of every four weeks while his leuprolide treatment was continued. His PSA level continued to rise exponentially for 18 weeks, thus we assumed docetaxel resistance. The patient revealed that he was ingesting a supplement of 10 daily dose units of Intrinsi B_12_/folate (Metagenics, San Clemente, CA, USA. Each dose unit contained 20 mg of porcine intrinsic factor and 500 μg of vitamin B_12_, as well as 400 μg of FA, and 400 μg of L-5-methyltetrahydrofolate (for a total of 800 μg of mixed FAs). On 11 February 2010, his PSA level reached 21.3 ng/mL, and on 25 February 2010, his serum FA level was assayed to be 134 ng/mL (normal range 5 ng/mL to 24 ng/mL), his serum vitamin B_12 _level was > 1500 pg/mL (normal range 300 pg/mL to 900 pg/mL), his serum testosterone level was < 20 ng/mL (normal range 212 ng/mL to 755 ng/mL), and his total serum homocysteine was 12.0 μmol/L (normal range 7 μmol/L to 12 μmol/L).

**Figure 1:**
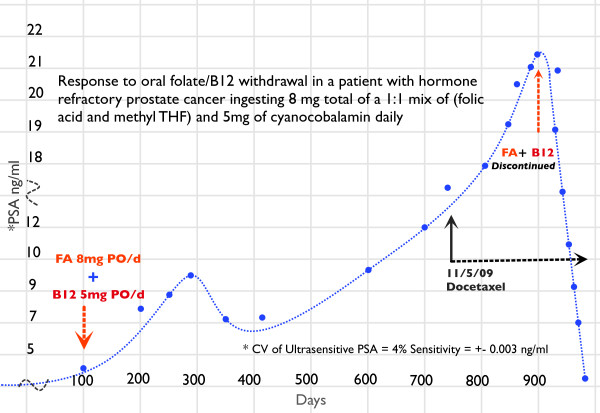
**The clinical course of our patient's prostatic-specific antigen response**.

The patient discontinued the oral supplement on day 900 (Figure 1), and within two weeks his serum PSA level started to decline. At the time of this writing, his PSA level is 2.08 ng/mL. He continues to receive weekly docetaxel chemotherapy. His last serum FA level was 4.0 ng/mL (borderline deficient), his serum vitamin B_12 _level was 377 pg/mL, and his total serum homocysteine level was 17.8 μmol/L.

## Discussion

**Figure 2:**
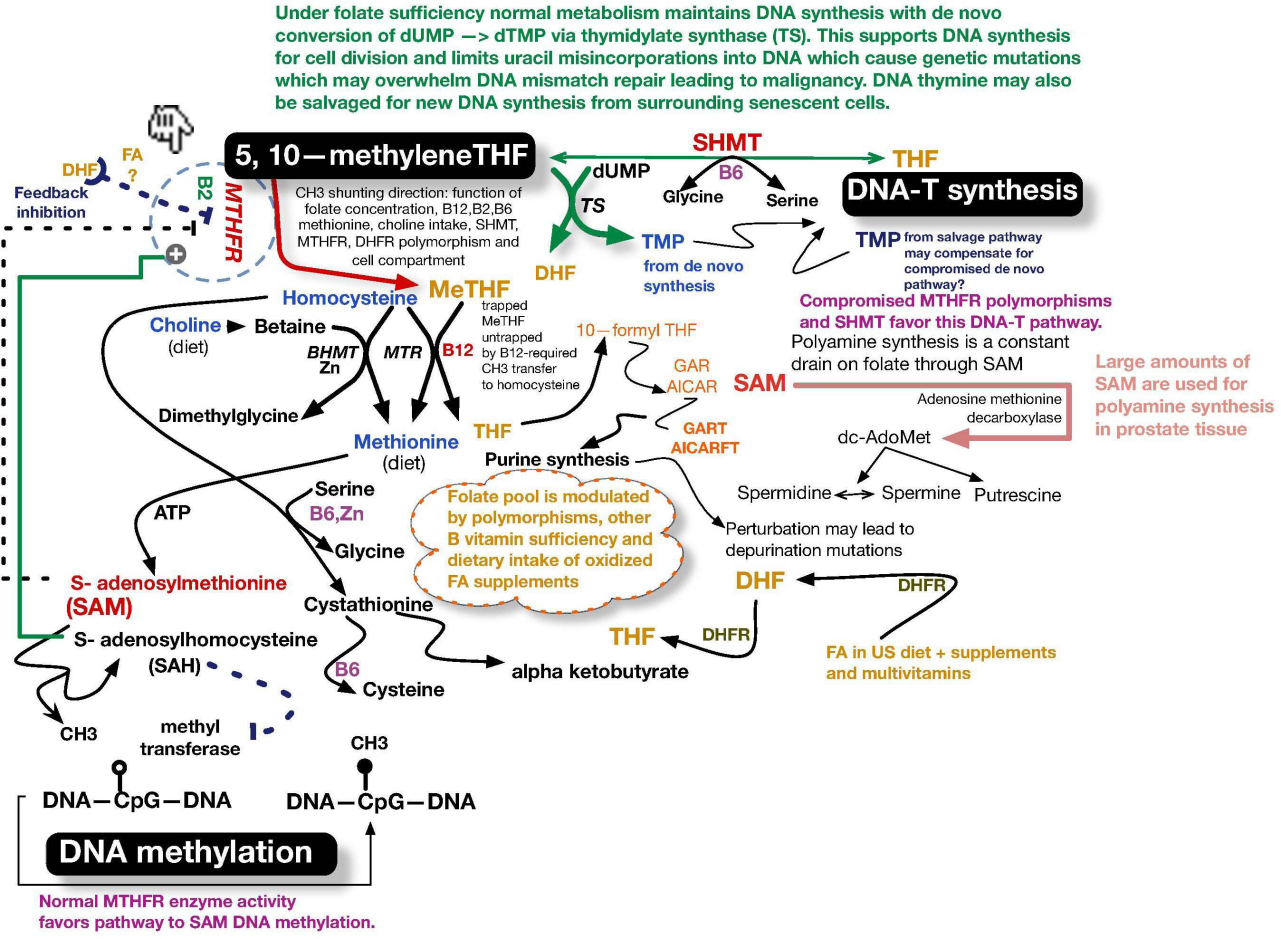
**Metabolic interactions between folates and vitamins B_12_, B_6_, and B_2_**. 677C → T thermolabile polymorphism with weakened interaction with B2 NAD cofactor disables MTHFR function by up to 70% in homozygotes. 15% of population is homozygous (2 inherited genes) 50% is heterozygous (one inherited gene). In the presence of this mutation (677C → T) when folate is plentiful this pathway provides adequate SAM for DNA methylation maintenance and shunts more 5,10 methylene THF to support DNA synthesis with less Uracil misincorporation into DNA with less 50% decreased incidence of colon cancer and acute lymphocytic leukaemia. However, in the presence of the mutation, if folate is low, then SAM DNA methylation may increase OR decrease and *de novo* DNA thymidine synthesis may decrease. There is disruption of normal intracellular methylated folate forms and all or some of these perturbations favour increased incidence of colon, breast, gastric, cervical and prostate cancer. Under most circumstances DNA synthesis through dTMP generation takes precedence over SAM DNA methlyation. Serine Hydroxymethyltransferase (SHMT) recently found to shift folate metabolism in the direction favoring *de novo* dTMP - DNA synthesis. B2 found to modulate (lessen) effects of MTHFR polymorphisms. Diet and all B vitamin levels modulate various folate pathways and therefore risks for malignancy!. Newly noted polymorphisms of DHFR (rs1677693 & rs1643659) have 30% decreased risk while MTR polymorphism (rs4659744) has 25% decreased risk of colon cancer only in the absence of FA supplements or FA supplemented diet. Changes in B vitamin concentrations and enzyme polymorphisms may produce unpredictable DNA methylation changes in part by varying DNA methyl transferase concentrations and SAM/SAH concentrations i.e. folate depletion may cause global DNA hypomethylation and specific CpG hypermethylations.

High-dose folate, FA, and vitamin B_12 _metabolic interactions may have modulated this patient's response to PSA treatment. Figure 2 summarizes folate and B vitamin biochemistry as they relate to the *de novo *and salvage pathways of DNA-thymine (DNA-T) and epigenetic regulatory effects of CH3 group transfer to the universal methylator *S*-adenosylmethionine (SAM) by FA and its vitamers.

### Some metabolic consequences of high-dose FA

Figure 2 illustrates the "untrapping" of L-methyl-THF by vitamin B_12_. This untrapping regenerates active reduced folate as tetrahydrofolate (THF). DNA-T formation is dependent on 5,10-methylene-THF, which passes its CH3 group to deoxyuridine monophosphate, thus forming thymidine monophosphate (TMP). TMP is subsequently phosphorylated, forming thymidine triphosphate, which is incorporated into DNA as DNA-T. In the absence of adequately reduced folates, uracil rather than thymine is incorporated into DNA, thus affecting DNA and its synthesis. Incorporated uracil leads to gene point mutations and may initiate malignant transformation. FA interference of dihydrofolate reductase (DFR) and polymorphisms of methyltetrahydrofolate reductase L-methyl-THF (MTHFR), that is, MTHFR 667TT, inhibits generation of 5-methyltetrahydrofolate reductase (5-methyl-THF). Pyridoxine (vitamin B_6_) exerts its influence in part through serine hydroxymethyltransferase (SHMT), the activity of which directs 5,10-methylene-THF in the direction of the *de novo *synthesis of DNA-T, thus minimizing the misincorporation of uracil into DNA.

FA is not naturally found in nature. In 1998, the US government mandated that the food supply be fortified with FA in an attempt to prevent neural tube birth defects. This action was associated with a tripling of the median normal serum folate level. However, FA has 3000-fold less affinity for DFR compared to dihydrofolate (DHF) [10], and its presence in high concentrations may induce steric interference, thus thwarting the reduction of natural DHF to THF and limiting the supply of reduced folates. In the USA, eating a normal diet while ingesting a multivitamin such as Centrum Silver (Pfizer Consumer Healthcare, Madison, NJ USA) is commonly associated with hypervitaminosis of FA to levels demonstrated to be associated with unnatural circulating levels of FA. In our practice, new patient serum folate levels usually exceed 25 ng/mL and are occasionally > 100 ng/mL. Ingestion of large amounts of FA affects the intra-cellular mix of folate vitamers from methyl-THF to non-methyl-THF [11,12]. Lucock and Yates [12] and others have proposed that the intra-cellular balance between the use of methylene-THF for DNA-T rather than for methionine synthesis may depend on the presence of both the MTHFR 677T polymorphism and high serum levels of FA. They noted that prolonged administration of large doses of FA is associated with greater reductions in intra-cellular concentrations of methylene, methenyl, formyl, and unsubstituted folate, while generation of vitamin B_12_-dependent, MTHFR-catalyzed methyl-THF levels decreased (Figure 2). The biological consequences of such a shift have not been thoroughly studied.

Other important metabolic interactions demonstrated by Smulders *et al*. [13] involving vitamin B_12 _and reduced folates included the folate and vitamin B_12 _dependence of the conversion of homocysteine to methionine minimizing toxic homocysteine while generating the universal methylator/epimethylator SAM. Changes in the SAM/*S*-adenosylhomocysteine (SAM/SAH) ratio due to changes in FA/folate concentrations, as well as the presence of hypersufficiency or insufficiency of vitamin B_12_, vitamin B_6_, and riboflavin (vitamin B_2_), may modulate the activity of folate vitamers. MTHFR and its many polymorphisms have profound effects as well [14,15]. High doses of both vitamin B_2 _and folates enhance the binding of the MTHFR co-factor flavin adenine dinucleotide (FAD) to MTHFR and its MTHFR 677T polymorphism. This FAD co-factor binding is weakened in the MTHFR (TT) and MTHFR (CT) polymorphisms, producing 60% and 30% less efficient heat-labile enzymes, respectively. The heterozygous MTHFR (CT) is present in about 40% of the US population, while two copies of the MTHFR (TT) allele are present in about 10% of the US population.

The concentrations of B vitamins and the presence of various coenzyme polymorphisms eventually affect gene expression and tumor behavior. Collin *et al*. found that higher serum folate levels are associated with increased risk [16] and faster progression [17] of localized prostate cancer.

### FA and epigenetic modifications in prostate cancer

The link between heritable epimethylation of cytosine bases within promoter cytosine-phosphate-guanosine (CpG) islands and cancer initiation, promotion, and progression is well established [18,19]. Its relevance to the genesis of prostate cancer is illustrated by methylation of the glutathione *S*-transferase (*GSTP1*) gene. Epimethylation of the *GSTP1 *gene is absent in normal prostate tissue and present in 6.4% of proliferative inflammatory atrophy, which is the precursor lesion of prostate cancer. *GSTP1 *hypermethylation is observed in 70% of patients with high-grade prostatic intra-epithelial neoplasia (a marker lesion associated with the subsequent development of prostate cancer) and in 90% of patients with prostate cancer [20].

In 2009, Figueiredo *et al*. [8] reported the results of administering 1000 μg of FA as a supplement to 327 men compared to 316 controls for approximately 10 years. Their controlled clinical study revealed an almost threefold higher incidence of prostate cancer in the group that received FA supplements (25 vs. 9 patients; *P *= 0.007 (logrank test) with an age-adjusted hazard ratio of 2.63). Hultdin *et al*. [21], in a study conducted in Sweden, observed that vitamin B_12 _supplementation was associated with an up to threefold increase in the risk of prostate cancer.

Yegnasubramanian *et al*. [22] noted that global DNA hypomethylation occurs later than CpG island hypermethylation in prostate carcinogenesis. These changes occur during prostate cancer progression and metastatic dissemination. Thus, DNA methylation may be responsible not only for carcinogenesis but also for tumor dedifferentiation as well as destabilizing genetic mutations, leading to tumor stimulation and metastasis. Collin *et al*. [16,17] found that two folate pathway polymorphisms (MTR 2756A > G and SHMT1 1420C > T) and circulating concentrations of vitamin B_12 _were associated with an increased risk of prostate cancer. Bistulfi *et al*. [23] demonstrated that prostate cells are highly susceptible to genetic and epigenetic changes caused by mild folate depletion.

E-cadherin, a transmembrane glycoprotein and a member of the cadherin family of cell adhesion molecules, mediates cell-cell adhesion via calcium-dependent interactions. E-cadherin, which may function as a tumor suppressor gene in tumor invasion and metastasis, is decreased or absent in many cancers and is predictive of tumor progression and poor patient outcome. In prostate cancer, decreased expression of E-cadherin correlates with hypermethylation of its promoter in patients' samples and human cell lines as well [24]. Pellis *et al*. [25] noted that incubation in the presence of high levels of FA (100 ng/mL) is associated with a marked decrease in E-cadherin expression in colon cancer cells *in vitro *[25].

As discussed above with regard to our patient, numerous possible FA and vitamin B_12 _pathways may have modulated prostate tumor growth. Ours is not the first patient with prostate cancer in whom we observed a response to a B vitamin [7]. We believe that the changes in PSA observed in our patient may have been related to changes in his serum levels of folate and/or vitamin B_12_.

Docetaxel therapy was deemed ineffective because of the absence of a PSA response during 18 weeks of administration. However, because the PSA decline occurred after withdrawal of B vitamins during docetaxel administration, the drug was continued for fear that there was a possible unknown relationship between it and the B vitamins. A search of the literature did not support a direct metabolic interaction between docetaxel or other taxanes and B vitamers; however, there is support for mediation of chemotherapy resistance to many drugs, including docetaxel, via DNA epimethylation [26-28]. We therefore consider the reversal of docetaxel resistance by folate/vitamin B_12_-mediated changes in DNA epimethylation to be possible. It is for this reason that our patient continues to receive docetaxel on the original schedule.

### Testosterone and folate metabolism

In the rat, castration caused marked changes in the content and distribution of various folate coenzymes in prostate tissue, which were reversed by testosterone replacement [29]. Castration caused suppression of the activity of prostatic DHF reductase (DHFR), a major rate-limiting enzyme, as well as 10-formyl-THF synthase and SHMT. Cytoplasmic SHMT acts in concert with vitamin B_6 _as a metabolic switch with at least three functions (Figure 2): (1) it preferentially supplies one-carbon units for DNA-thymidylate synthesis by favoring the conversion of glycine to serine, (2) it lowers methylene-THF used for SAM synthesis by preferring serine synthesis, and (3) it essentially sequesters 5-methyl-THF, thus sacrificing SAM synthesis [30]. The administration of testosterone restored the enzymatic activities to close to normal values. First described by Rovinetti *et al*. in 1972 [29], these castration-related changes, if present in human prostate tissue, could produce powerful metabolic and genetic changes modulated by the testosterone level of the patient.

There are several metabolic inter-relationships between FA and its vitamers aimed at the shuttling of methyl (CH3-) groups to support the synthesis of DNA-thymine and to deliver methyl groups to the universal methylator SAM. The other B vitamins, B_2_, B_6_, and B_12 _plus methionine, a diet-supplied amino acid, as well as choline (eventually metabolized to methyl groups), support methyl group generation and folate metabolism. SAM delivers the methyl groups responsible for promoter CpG islands and global DNA cytosine epimethylation. Epimethylation of a gene promoter region switches off transcription of the gene's exon(s). Exon transcription yields mRNA synthesis which will eventually lead to synthesis of regulatory proteins/enzymes.

## Conclusion

Our patient's clinical course suggests that high doses of B vitamins (FA/folate and vitamin B_12_) may modulate the course of PSA failure in castrate-resistant/refractory prostate cancer. Our patient's ingestion of large amounts of FA/folate and vitamin B_12 _was associated with PSA acceleration, while withdrawal of the supplements was associated with a significant PSA decline. Whether this result was secondary to the perturbation of the outlined metabolic interactions of B vitamers (Figure 2), due to DNA epimethylation with associated changes in gene expression, or due to other factors is unknown. The hypothesis of a yet to be discovered interaction with docetaxel is entertained as well. Studies of patient use of health store supplements, many of which are known to affect DNA metabolism and DNA methylation markers, have revealed that up to 50% of cancer patients ingest large doses of vitamins and other supplements, such as probiotics, which contain "safe" bacteria that generate copious amounts of folates within the bowel. A recent study in our clinic revealed that a majority of cancer patients present with hypervitaminosis or hypovitaminosis of at least one or more of the B vitamins noted in Figure 2. As we have discussed herein, castration, drugs, diet, vitamin supplements, and probiotics may modulate tumor cell metabolism as well as gene expression by epimethylation and synthesis of DNA. We are concerned about the finding that many gas stations and liquor stores in the USA sell so-called "quick energy" liquid supplements that contain large amounts of B vitamins, including vitamins B_6 _and B_12 _and FA.

### Patient's perspective

"I was alarmed to see a steady increase in my PSA while undergoing chemotherapy. I had been taking a dietary supplement containing large amounts of vitamin B_12 _and folate. When I learned of the relationship of large amounts of folate to increasing PSA, I immediately stopped taking the supplement. My wife and I searched the Internet to find foods that were low in natural folate, and avoided those foods high in folate. When I found out the government had mandated the addition of folic acid to basically all products containing flour and grains since 1998 I was surprised and dismayed. We quickly learned to read the food content labels on packaging, and found that many foods contained folic acid. Avoiding folic acid supplemented foods meant that nearly all baked goods, cereals, crackers, pasta, egg noodles, stuffing mix, white rice, bagels, flour tortillas, sandwiches, burger rolls, dinner rolls, doughnuts, pizza, pies and cakes were off limits. My wife, who loves to bake, found organic flour that was not supplemented, and used that exclusively to make bread, cookies, pies, cakes, pizza dough, etc. Happily, doing something as simple as reducing FA intake, allowed the chemotherapy to rapidly reduce my PSA to the lowest level in years."

## Consent

Written informed consent was obtained from the patient for publication of this case report and any accompanying images. A copy of the written consent is available for review by the Editor-in-Chief of this journal.

## Competing interests

The authors declare that they have no competing interests.

## Authors' contributions

GT and AG analyzed and interpreted the data regarding the patient's clinical course, therapy, ingested supplements, and laboratory vitamer and PSA response. GT was a major contributor to the writing of the manuscript. Both authors read and approved the final manuscript.
